# Performance of Poly(lactic acid) Surface Modified Films for Food Packaging Application

**DOI:** 10.3390/ma10080850

**Published:** 2017-07-25

**Authors:** Valentina Siracusa, Marco Dalla Rosa, Alexey L. Iordanskii

**Affiliations:** 1 Department of Chemical Science, University of Catania, Viale A. Doria 6, 95125 Catania (CT), Italy; 2 Interdepartmental Centre for Agri-Food Industrial Research, Alma Mater Studiorum, University of Bologna, Piazza Goidanich 60, 47521 Cesena (FC), Italy; marco.dallarosa@unibo.it; 3 Semenov Institute of Chemical Physics, Kosygin str. 4, 119991 Moscow, Russia; aljordan08@gmail.com

**Keywords:** biodegradable food packaging, structure-property relations, mechanical properties, gas barrier behavior, poly(lactic acid)

## Abstract

Five Poly(lactic acid) (PLA) film samples were analyzed to study the gas barrier behavior, thermal stability and mechanical performance for food packaging application. O_2_, CO_2_, N_2_, N_2_O, and C_2_H_4_ pure gases; Air; and Modified Atmosphere (MA, 79% N_2_O/21% O_2_) were used to analyze the influence of the chemical structure, storage temperature and crystalline phase on the gas barrier behavior. The kinetic of the permeation process was investigated at different temperatures, ranging from 5 °C to 40 °C. Annealing thermal treatment on the samples led to the crystalline percentage, influencing especially the gas solubility process. Thermal properties such as T_g_ and χ_c_, and mechanical properties such as tensile strength and modulus were remarkably improved with surface PLA modification. A more pronounced reinforcing effect was noted in the case of metallization, as well as improved gas barrier performance. Tensile testing and tensile cycling tests confirmed the rigidity of the films, with about a 20% loss of elasticity after 25 cycles loading.

## 1. Introduction

Currently, the high-technology treatments such as low-storage temperature and Modified Atmosphere Packaging (MAP) are commercially used to reduce the food respiration rate, delaying senescence, and, ultimately, increasing in the packaged food shelf-life [1,2] The main objective of this technology applied to the food preservation is the reduction of respiratory activity, delay in softening and ripening, reducing incidence of various psychological disorders and pathogenic infestations. MAP is used to alter, in a controlled way, the gaseous composition inside the package through passive or active kinetic mechanisms. Two processes are responsible for achieving this positive result: the respiration rate control of the food product and the gas transfer through the packaging material. Both phenomena are related to each other and depend on many factors, such as packaging structure, barrier thickness, gas pressure, and temperature. In particular, metabolic processes such as respiration rate, endogenous fermentation, and film permeability increase with the temperature. Therefore, when selecting an appropriate packaging material, among different characteristics (material flexibility, machinability, clarity, durability, resistance to chemical and thermal degradation, etc.), the gas barrier feature is one of the most important for food application. 

The main permeates used in MAP technology are O_2_, CO_2_ and N_2_, at different levels. The O_2_ concentration is responsible for the food respiration rate. The decrease in food respiration rate delays enzymatic degradation, extending the shelf-life of the product. However, if an excessively low O_2_ level occurs, tissue deterioration could develop, leading to off-flowers and off-odors production [2]. CO_2_ confers a significant level of antimicrobial influence on the food packaged. The CO_2_ influence was well explained by Farber [3] in his review. N_2_ is an inert gas used for mimicking completely the inside package atmosphere, preventing also the film collapse. In his review, Caleb [2] has comprehensively summarized selected review publications on MAP progress applications. 

Recently, there has been a great interest in the potential benefits of other non-conventional gases such as the noble gases (Ar, He, and Xe) and nitrous oxide (N_2_O) [4]. Although these gases have been successfully used to preserve vegetables and fruit, their commercial use requires further coherent research. Several studies have found that they exert an effect on the metabolic activity of various vegetables through unknown mechanisms. As reported by Rocculi [5], inconsistent results have been achieved by Ar inhibition and control of degradation reactions in perishable fruit products. Silveira [4] deals with the inability of this gas to inhibit the microbial growth in fresh products such as broccoli, lettuce and arugula. Ar acts selectively enhancing the diffusion of gases such as CO_2_ and C_2_H_4_ from plant tissue rather than N_2_. Similarly, He increases the O_2_ diffusion, reducing the risk of fermentation. N_2_O, a “new packaging” gas allowed for food use in the EU, seems to perform well by reducing the fruits and vegetables tissue respiration and ripening, inhibiting the ethylene synthesis, prolonging the shelf-life of Modified Packaged (MP) fresh food, and increasing the storage life [4,5]. Regarding the C_2_H_4_ gas, until now no data exist in the literature on the barrier behavior of packaging plastics films. However, this gas promotes the enzymatic activities, softening and ripening of fresh food such as cut fruit and vegetables. It is responsible for increased chlorophyllase activity that causes chlorophyll destruction and its conversion to the olive brown pheophorbide, with changes in chromatic characteristics of the vegetable tissue [5]. In order to prevent the accumulation of this gas inside the package, an ethylene absorbent could be used, but knowing the polymer gas barrier performance with C_2_H_4_ at different storage temperature could be a good further alternative to maintain the quality of packed food [5]. 

In the last fifteen years, particular interest and attention have been paid to the polylactic acid polymer (PLA), especially when it is used as packaging materials for products being sensitive to environmental conditions, such as food or pharmaceutical goods [6,7,8,9,10,11]. PLA is the most commercial biodegradable polymer, showing the advantage to easy change and tailoring its physical and mechanical properties by simply changing the chemical composition (amount of d- and l-isomer) and the processing conditions. Hence, different methods have been suggested in order to improve its chemical-physical performance. Concerning the barrier behavior, in respect to the synthetic polymers, it shows a high gas transmission that renders this polymer not suitable for several food packaging applications. To improve the gas barrier characteristics, the surface polymer modification such as coating and metallization could be performed [12,13]. Especially, the barrier properties and the tensile strength can be improved by applying such operations [10]. Generally, metallization is used to enhance the barrier characteristics and, in addition, to provide an essentially total light screen [12]. The transport phenomena background followed in this work is well described in the literature, with a comprehensive description of the mathematical equations and corresponding interpretation [14,15].

The general aim of this investigation is to study the effect of the use of conventional and non-conventional gas and gas mixture on the barrier performance under different storage temperature, for the biodegradable material, PLA. Specifically, we used five modified and unmodified PLA films:

- to evaluate the principal parameters of gas transmission rate (GTR) such as solubility (S), diffusivity (D) and time lag (t_L_) for several penetrants, namely atmospheric gases (O_2_, CO_2_, and N_2_), unconventional gases (N_2_O and C_2_H_4_), and gaseous mixes (Air and Modified Atmosphere (MA));
- to analyze the correlation between PLA film structure and gas type used in the experiment;
- to assess the temperature dependence of GTR and perm-selectivity coefficients through the determination of the corresponding activation energies; and
- to correlate the PLA barrier behavior with its thermal and mechanical properties including cyclic stress-strain measurements.

## 2. Results and Discussion

### 2.1. Barrier Behavior

Gas transmission in polymer materials depends strongly on the polymer structure, on the gas type, as well as on the temperature. The theoretical models that have been proposed and reported in the literature [16,17] describe the transport mechanism of molecular species in polymers by diffusion, involving a temperature-dependent diffusion coefficient. However, it should be observed that it is not easy to identify this dependency exactly and to provide exact information on both the diffusion coefficient and the solubility coefficients, in particular under real condition [18,19,20]. Therefore, in-depth analyses of the relationship between such parameters are needed to get the best market solution and to enable better understanding of the underlying transport phenomenon that occur in real conditions. The primary mechanism for gas flow through a film or coating is an activated diffusion. This means that the permeate dissolves in the film matrix at the higher concentration side, diffuses through the film, driven by a concentration gradient and evaporates from the other surface. Differences in the solubility of specific gases may influence the diffusivity of gases across the film. The second step of the permeability phenomenon, the diffusion, depends on the size, shape and polarity of the penetrating molecule of the permeant and on the crystallinity, degree of cross-linking and polymer chain segmental motion of the polymer matrix. Permeability of polymers to an organic compound or water is presented using the Gas Transmission Rate (GTR), which is in common use as well just reported in literature [12,21,22].

In our work, we correlate the GTR values of five PLA films with the main influencing factors such as temperature, gases and chemical structure. GTR values (cm^3^/cm^2^ d bar), together with Solubility S (cm^3^/cm^2^ bar), Diffusivity D (cm^2^/s) and time Lag t_L_ (s), were recorded for pure gases, Air and MA, together with thickness (in micron) and perm-selectivity ratio. In Table 1 are reported all the collected data recorded with O_2_ gas test, at 23 °C, while in Table 2 are reported the corresponding perm-selectivity data, recorded with all gases used in the experiment and at all temperature investigated (the value recorded at 23 °C is in bold style to be used as standard value). 

Perm-selectivity values represent the permeability ratio of different permeants As reported by Schmid et al. [23], for many polymers, this ratio is in the range 1:4:16 (N_2_:O_2_:CO_2_). The calculated perm-selectivity ratios are shown in Table 2. The results are very different from the simplified ratio. These data are useful because they give the possibility to determine the unknown GTR data, knowing the GTR value of another gas. For example, obtaining the CO_2_ GTR data is enough to multiply the O_2_ GTR data for the corresponding perm-selectivity ratio, at the desired temperature. It will be an error to use the tabulated values present in the literature because it was scientifically demonstrated that the perm-selectivity ratio are not constant for every type of polymer materials but it changes depending on several factors, first of all the chemical structure and experimental temperature [23,24,25].

As expected, the surface modification effectively retarded the transport of gas molecules through the PLA matrix, resulting in a substantial decrease in GTR value. PLA samples with larger amount of oxygen-containing functional groups were found to yield more prominent improvement in barrier performance, giving rise to better compatibility with PLA resins, ultimately resulting in effective suppression of gas molecule permeation through the films.

CO_2_ and N_2_O gases were more permeable through the polymer film, with respect to O_2_ and N_2_, despite their different molecular diameters being larger than of for oxygen and nitrogen molecules. The C_2_H_4_ gas transmission is quite low, very similar to the N_2_ gas. GTR behavior in a modified atmosphere (Air and MA) is instead strictly dependent from the superficial treatment of the PLA film. It is well known that the mechanisms that drive the adsorption/desorption permeability, solubility and diffusion phenomena are all closely dependent on the temperature [21,22] so, as expected, temperature has a considerable influence on the transmission of the gas through the material. Considering polymeric film, which performs optimally at a given temperature, may increase/decrease the rate of food respiration or material permeability at a higher/lower temperature, it is important to understand the PLAs gas barrier performance under a range of temperature generally used for food storage. In fact, limited information is still available on most film’s permeability properties at varying storage temperature, as well as on the ability of the polymeric film to withstand mechanical stress during storage and transport. Gas transmission behavior was then described in detail.

**Figure 1:**
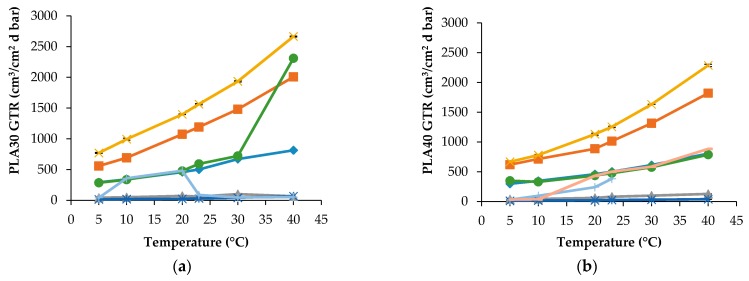
GTR at different temperature, with different gases: (**a**) PLA30 film (♦ O_2_, ■ CO_2_, **Δ** N_2_, **✕** N_2_O, * C_2_H_4_, ● MA, **+** Air) and (**b**) PLA40 film (♦ O_2_, ■ CO_2_, **Δ** N_2_, **✕** N_2_O, * C_2_H_4_, ● MA, **+** Air_1_, **-** Air_2_).

In Figure 1 GTR data for PLA30 and PLA40 films obtained at different temperature and for various gases are reported. 

Both samples have differences in thickness and crystalline percentage, therefore their barrier characteristics differ each other due to change in the gas diffusion pathway and the amorphous state in the intercrystalline area of PLA films. For the PLA30 specimen, the transport of CO_2_ and N_2_O has a similar behavior, however N_2_O is characterized by higher gas transmission in comparison with CO_2_. If compare the transfer rates of principal atmospheric gases, such as O_2_ and N_2_, the former has essentially higher GTR than the latter. Additionally, it is worth noting that C_2_H_4_ exhibits GTR values practically similar to the N_2_ values. Especially, the difference in transport behavior is noticeable for the modified atmospheric composition of the gases (MA). In this situation, the temperature dependences of GTR for both PLA samples displays the different forms, namely the curve with a sharp inflection point at 30 °C for PLA30 and the monotonic temperature curve for PLA40. Besides, Figure 1 shows that the MA-GTR values are close to the O_2_ values in whole temperature interval for the PLA40 film but for the PLA30 film the given proximity occurs only by 23 °C. The analogous comparison between MA and N_2_ permeabilities shows that, for both specimens, while for N_2_ the analogous behavior is observed at T < 23 °C and only for the PLA40 film. Qualitatively, the analogous domination in MA-GTR is observed for N_2_O transferring. The next step in estimation of gaseous mix behavior represents the measuring of air permeability. In Figure 1b, the transfer behavior of air is reported as: Air_1_ (the first 10 minutes of GTR measurement) and Air_2_ (after 10 minutes of GTR measurement). In other words, there are short and long terms of measurement. In Figure 1b, Air_1_-GTR values for the PLA40 film are increased until 23 °C. The Air_2_-GTR values reach constant values similar to N_2_ and MA, respectively. The quantitative description of MA transport and its comparison with the single gas transport seems to us extremely important because similar modeling brings above results closer to the real conditions when using eco-friendly PLA packaging.

**Figure 2:**
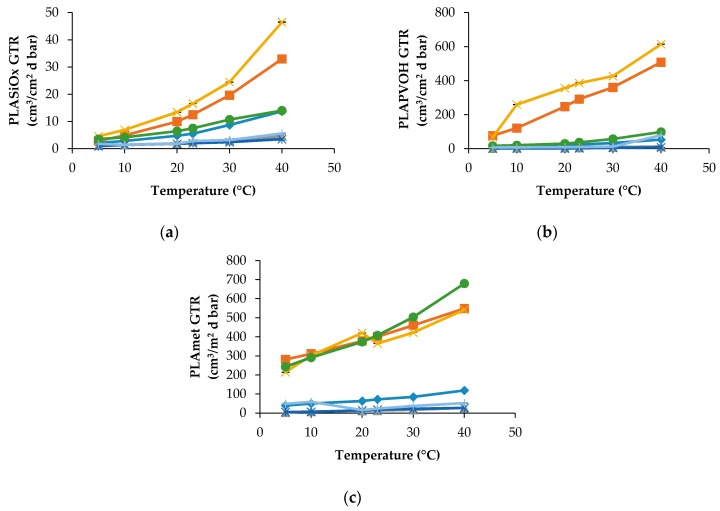
GTR at different temperature of different gases (◆ O_2_, ∎ CO_2_, **Δ** N_2_, **✕** N_2_O, * C_2_H_4_, ● MA, **+** Air) for: (**a**) PLASiO_x_; (**b**) PLAPVOH; and (**c**) PLAmet.

PLA modified films show a very different trend. As can be observed in Figure 2a, the PLASiO_x_ specimen displays the best barrier characteristics for all penetrants including the pure gases and MA.

At all temperatures, the gaseous mixtures, such as MA and Air, have trends and GTR values that are quite close to O_2_ GTR and N_2_ GTR, respectively. Even when the multicomponent penetrant is applied, its transport phenomenon could be approximated by a simple gaseous penetrant what facilitates the prognosis of barrier properties of biopolymers.

PLAPVOH film shows a dual behavior, being dependent on the chemical nature of gaseous penetrant. CO_2_ and N_2_O show the higher GTR value relatively to the other gases (C_2_H_4_, N_2_ and O_2_) and modified atmosphere (Air and MA). Analogous results were obtained for N_2_ gas and Air at all temperatures, while a small GTR difference was observed between O_2_ gas and MA atmosphere at temperatures up to 20 °C.

PLAmet showed a different behavior. GTR recorded for CO_2_, N_2_O and MA demonstrate the same tendency with higher recorded value, while GTR data obtained for C_2_H_4_, O_2_, N_2_ and Air are characterized by lower GTR value. 

In general, superficial treatment lowered the GTR value, especially when SiO_x_ was used as a coating. 

**Figure 3:**
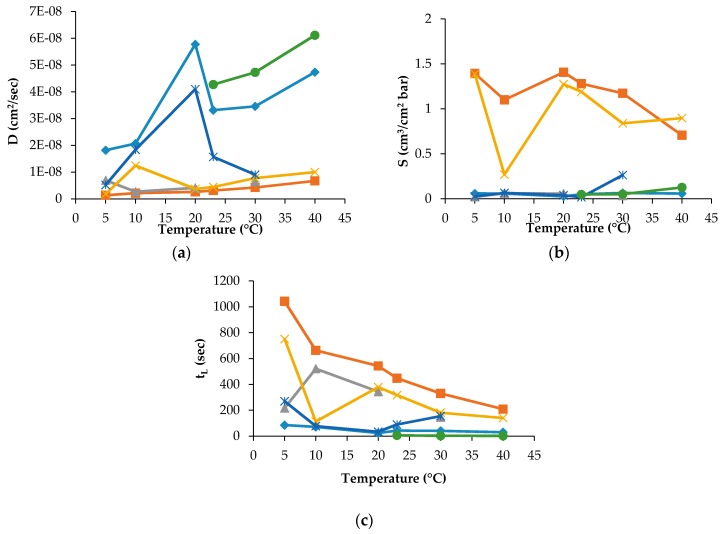
Permeability coefficients (**a**) D; (**b**) S; and (**c**) t_L_ for PLA30 sample (♦ O_2_, ∎ CO_2_, **Δ** N_2_, **✕** N_2_O, * Air, ● MA).

**Figure 4:**
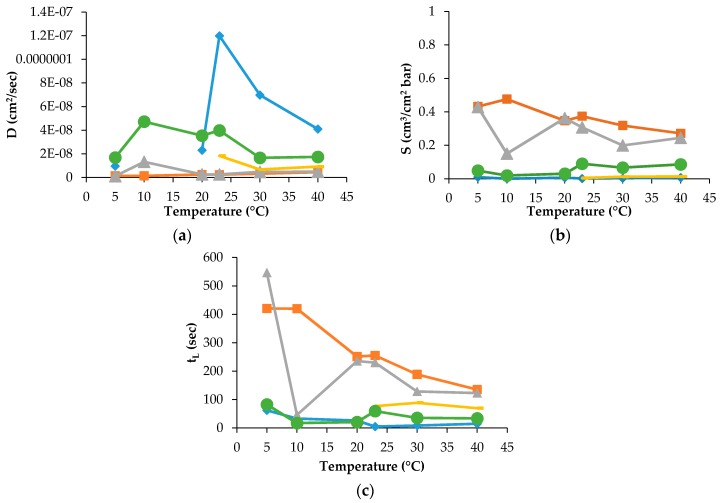
(**a**) D; (**b**) S; and (**c**) t_L_ for PLAmet sample (♦ O_2_, ∎ CO_2_, **Δ** N_2_O, **-** Air, ● MA).

The D, S and t_L_ data were recorded for PLA30, PLA40 and PLAmet samples, but not for all gases and temperature employed in the experiment. For PLAPVOH, it was possible only with CO_2_ and N_2_O gases, while, for PLASiO_x_, no data were recorded because it was not possible to fit the slope of the linear portion of the GTR curves [26]. The D, S and t_L_ are reported in Figure 3 and Figure 4 for PLA30 and PLAmet, respectively.

The other data recorded for the other samples were not reported for the sake of simplicity.

The D value, correlated to the kinetic parameters, showed a similar trend for all gases. This means that the diffusion was the same into the film for all tested gases and gas mixture. PLA coated films showed a high barrier behavior because of a very low diffusion coefficient. The surface modification decreased the gas transmission through the films thanks to the reduction of the gas diffusion [13,27]. 

The effect of temperature on the solubility coefficient differs depending on the chemical structure of penetrant. S values of used gases increased according to the following order: N_2_ < O_2_ = Air = MA < CO_2_ < N_2_O, in the range of temperature investigated. The oxygen-containing composite molecules of gases (N_2_O and CO_2_) have higher thermodynamic affinities to the polymer segments with the ester groups. Simultaneously, the lowest equilibrium solubility and, respectively, the small thermodynamic affinity belongs to N_2_ and O_2_ molecules. The intermediate position is occupied by the gas mixtures on the base of molecular oxygen enriched with molecular nitrogen (MA) and Air. Thus, the gas permeability of the PLA films as the product of the diffusion mobility and solubility determined by the size of the diffusing particles (on the kinetic level) and the gas equilibrium sorption in polymer (on the thermodynamic level). The latter is necessary for the determination and recording of the boundary conditions for a correct solution of a Fick diffusion equation and gives its own independent contribution to the overall gas transport.

According to a widely known classical diffusion–solubility model [26,27] of gas permeability through polymer films and membranes, gaseous penetrant solubility determine the mathematical border conditions of the principal diffusivity equation (Fick’s diffusivity) describing the diffusion impact upon gas permeation. Furthermore, the GTR is generally determined by the product of gas diffusivity (D) by its coefficient solubility (S). Therefore, gas solubility evaluation in PLA and PLA modified films with different crystallinity and morphology provides the key information on barrier characteristics so essential for packaging. 

The t_L_ values as the basic experimental parameters required for gas diffusivity evaluation correlate with the GTR values: as GTR meanings are increased, t_L_ meanings are decreased in accordance with the relation

t_L_ = kL^2^S/6[GTR], (1)

where L is the film thickness, k is the numerical coefficient reflecting the dimension of GTR, and S is gas coefficient solubility. For PLA30, the t_L_ values are decreased with temperature increasing from the highest values at 5 °C from ~1350 s to ~750 s for CO_2_ and N_2_O, respectively, to the lowest ones at 40 °C for the same gases. The t_L_ values of O_2_, N_2_, Air and MA have a sufficiently weak trend of decrement ranging from 85 s to 40 s, from 216 s to 145 s, from 269 s to 154 s and from 33 s to 22 s, respectively. Analogous tendency is observed for the PLA40 specimen where the highest values were recorded at 5 °C for CO_2_ (780 s), for N_2_ (700 s) and for N_2_O (1650 s). For O_2_, Air, and MA the t_L_ decrease is observed as well, when the corresponding values range from 211 s to 97 s, from 60 s to 29 s, 106 s to 84 s, respectively. For PLAPVOH, the highest value was observed at 5 °C for CO_2_ (1852 s) and N_2_O (2629 s). For PLAmet, the highest value was recorded at 5 °C for CO_2_ (420 s), for N_2_O (546 s), while, for O_2_, Air and MA, were quite constant, ranging from 62 s to 26 s, from 77 s to 69 s, from 82 s to 59 s, respectively.

### 2.2. Activation Energy of Gas Transport Process

**Figure 5:**
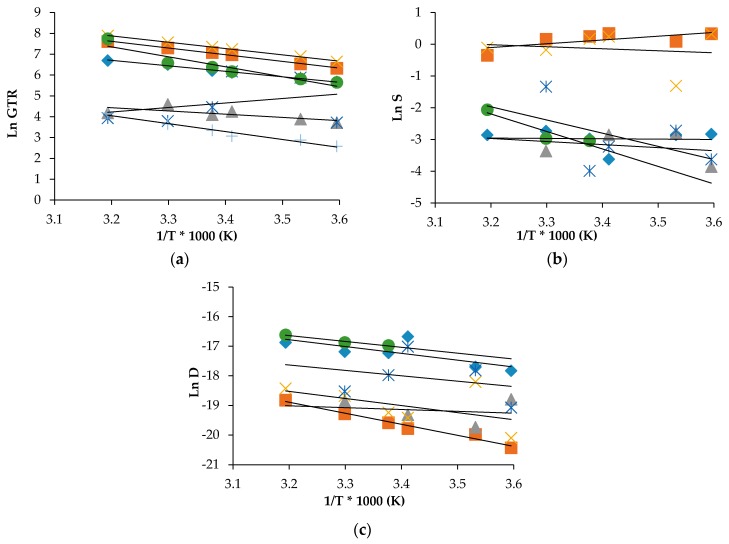
Arrhenius plot coefficients for: (**a**) GTR (♦ O_2_, ∎ CO_2_, **Δ** N_2_, **✕** N_2_O, * Air, ● MA, **+** C_2_H_4_); (**b**) S (♦ O_2_, ∎ CO_2_, **Δ** N_2_, **✕** N_2_O, * Air, ● MA); and (**c**) D (◆ O_2_, ∎ CO_2_, **Δ** N_2_, **✕** N_2_O, * Air, ● MA), for PLA30 film.

In order to describe the permeation dependence to the temperature, the Arrhenius model was utilized to calculate the activation energy for gas transmission (E_GTR_), heat of solution (H_S_) and diffusion (E_D_) processes. The mathematical relations used are well described in the related scientific literature [15,19,23]. The activation energy is obtained by calculating the value of the slope (−E_a_/R) of the Arrhenius equation, where R is the gas constant (8.314 J/mol K). Natural logarithmic (ln) of GTR, S and D compared with reciprocal of the absolute temperature (1/T) were reported as an example in Figure 5 for PLA30, together with the indication of the calculated linear regression of the corrected experimental points fittings. 

Table 3 and Table 4 contain the corresponding energy characteristics such as activation energies for the gas transmission rate (E_GTR_), the specific heat of gas solution (H_S_) and activation energy of gas diffusivities (E_D_) in the temperature range of 5 °C–40 °C. 

As can be observed, the data satisfactory fitted the theoretical relation with the high values of regression coefficient (*R*^2^) that indicates a good linear correlation between permeability and reciprocal temperature for all simple gases. Practically all individual gases and mixes, excluding Air-PLAmet system, have the negative slopes of the semilogarithmic temperature function of GTR that correspond to the positive meanings of E_GTR_ for both pristine and modified PLA films (see the respective data in Table 3 and Table 4).

The corresponding E_GTR_ characteristics have the highest values for N_2_O and CO_2_ gases containing oxygen in the molecular formula as compared with the permeability characteristics of unpolarized gas molecules such as C_2_H_4_, N_2_ and O_2_. When the gaseous mixes such as Air and MP are used, different tendencies for GTR, S and D are observed, depending on the way of modification of PLA. For the solution and diffusion coefficients some deviations were observed. According to N_2_O and CO_2_ gas test data, the solubility increased with the temperature while N_2_ and O_2_ solubilities were almost constant. Simultaneously, for MA and Air, there is not the linear trend that confirms the difficulty in measuring the permeability of modified atmosphere as the multicomponent medium.

Generally speaking, gas solubility is the parameter correlated to the polymer composition so this trend is a confirmation that the gases interact differently with the matrix. From low to high temperature, the lnS value shifts with a scissors trend, and the corresponding H_S_ shows a fluctuating value, positive for N_2_O and CO_2_ gas test and negative for the others, comprising Air and MA. LnD shows a standard behavior with all gases and mixture, thus meaning that the diffusion was the same for the tested gas, despite the different solubility. Consequently, E_D_ shows a positive value for all pure gas tests but not for modified atmosphere (Air, MA). Similar results were recorded for the PLA40 and PLAmet samples. For PLASiO_x_ sample, no S and D data were recorded, while, for PLAPVOH sample, only few data with CO_2_ and N_2_O gases were recorded (data not reported for the sake of simplicity). As it is well known from the literature [28,29,30], high activation energies imply more sensitivity to temperature variation. The permeation process is very well correlated to the temperature variation, while the sorption/diffusion process shows consistent deviation, more correlated to polymer structure. Further, the corresponding selectivity ratio shows different value depending on the temperature, confirming that also this parameter is not a constant and is correlated not only with the chemical structure of the materials, but depends also from the (analysis) temperature. Values were reported in Table 2. The behavior could be explained with the crystalline percentage calculated after annealing of the sample. As the crystalline phase increased, the E_GTR_ and E_D_ increased while the H_S_ decreased. It means that, if the gas transmission increases (less barrier behavior), the solubility increase (major interaction with the polymer matrix) and the diffusivity decrease (the gas molecules spend less time in moving inside the polymer matrix). Consequently, if E_GTR_ decrease (less energy required for the gas transmission process), H_S_ decrease (the gas solubility is enhanced) and E_D_ increase (more difficult by the gas molecules to move inside the polymer matrix). This trend varies with gas and temperature and underlain the importance to perform the determination of the barrier characteristics at different storage temperature.

### 2.3. Thermal Behavior

**Figure 6:**
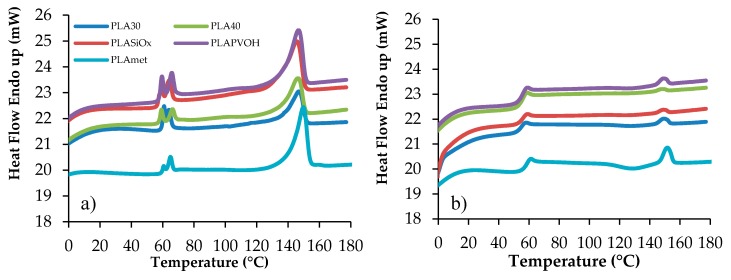
Calorimetric curves of PLAs films: (**a**) first scan; and (**b**) second scan from the melt.

Samples have been stored at ambient temperature for 30 days in order to provide the same heat treatment for all the samples investigated, erasing their previous thermal history. In order to calculate the PLA crystalline degree in percentage, the following formula was used: x_c_ = 100 ((ΔH_m_ − ΔH_c_)/ΔH_m_*), where ΔH_m_ is the fusion enthalpy calculated from the experimental DSC curve, ΔH_c_ is the crystallization enthalpy calculated from the experimental curve as well, and ΔH_m_* = 93 J/g is the fusion enthalpy for the entirely 100% crystalline PLA [13]. The corresponding Differential Scanning Calorimetry (DSC) curves for the neat and surface-modified specimens of PLA are represented in Figure 6. Using these experimental data, the principal thermal transition characteristics are collected in Table 5.

T_g_ value was not observed from the first heating run due to the presence of the crystalline phase, the corresponding calorimetric traces characterized by a conspicuous melting endotherm. Although PLA is an ideal polymer for environmental application, it has some disadvantages such as high crystallinity and consequently high brittleness, at ambient temperature. This T_g_ was relatively weak and difficult to determine. Furthermore, it can be seen from the first heating run that the PLA film is a multilayer material, showing a first transition phase, represented by two endothermic peaks, corresponding to the fusion of the polymer substrate material on which PLA is added [13]. The first two T_m_ values, represented by endothermic phenomena, are so correlated to the substrate polymer film behavior and are recorded in the range where the T_g_ is detected. In order to detect the T_g_, an amorphous polymer was obtained by rapid cooling from the melt. In this case, during the second heating run, a consistent transition was detected, associated to the glass-to-rubber transition, with the corresponding Δc_p_ value, with a small endothermic peak corresponding to a residual crystalline PLA, with a T_m_ value slightly higher due to more perfect crystallites.

As can be observed, the T_g_ value varies from 54 °C to 58 °C and the melting temperature is in the range from 145 °C to 150 °C value, in accordance with the results reported in literature [29]. To determine the possible change in PLA crystallinity during the gas transfer measurements, the DSC curves were recorded for the samples being stored for a different time. Under isothermal conditions, the storage time was chosen on the base of GTR measurement duration, namely from 1 h to 4 h (see Table 6). Crystallinity percentage for the uncoated and coated films is weakly dependent on the temperature of gas permeability, while the way of surface modification alters the PLA crystallization by about 30%. After quenching and DSC rescanning, the degree of crystallinity was very small evidencing the low ability of PLA to recrystallize during cooling, except for PLA metallized sample. As reported also from Kim and Choi on its paper on PLA/graphene films [30], modification of neat PLA matrix was found to increase the T_g_ value. Such a reinforcing effect in the thermal property may be ascribed to the reduced mobility of PLA chain molecules in the modified materials, resulting from the interaction between the surface ester groups of the polymer molecules and the groups of modifying layer. Similar results have likewise been reported for a number of nanocomposites incorporated with various nanofillers such as nanosilica particles, nanoclays, cellulose nanofiber and graphene nanosheets [30,31,32,33,34,35], and PLA blend with other polymers (biodegradable or not) [11]. It should be noted that the metallization led to a more substantial increase in T_g_ of the PLA composite, compared to neat PLA, because the metallization could yield more interaction between the two phases, leading a more homogeneous composite material. As regards crystallization behavior, the χ_c_ of PLA in the modified films was revealed to slightly change with the exception of the metallized PLA. It could be seen that the incorporation of a metallized layer led to a remarkable increase in χ_c_. The aluminum incorporated into the PLA matrix may be considered as extraneous nucleating agents promoting the crystallization rate, and thus yielded the noticeable improvement in the degree of crystallinity for PLA film. Additionally, the PLA metallized exhibited a more pronounced effect on the crystallization behavior compared to the other films, which may be associated with uniform dispersion in the PLA matrix. The crystallization process acceleration caused by the incorporation of fillers has also been reported in a variety of crystalline polymer-based nanocomposites [36,37].

In order to understand if during the gas barrier measurements, performed at different temperature, the crystallinity of the sample changed, influencing consequently the barrier behavior measurements, DSC scans were recorded after 1 h to 4 h of sample maintained in isothermal conditions (annealing treatment) at different temperatures. The isothermally keeping time of the samples was chosen considering the time required to obtain a representative GTR curve in its equilibrium state (steady state), with the different gases and at different temperatures. Data recorded are reported in Table 6.

As can be observed, a different crystalline percentage was recorded, confirming that temperature plays a key role in the final performance of the tested films. The crystallinity changes are different, depending on the PLA film type. The influence of change into the crystalline/amorphous phase was much more evident in the solubility coefficient value (S). Therefore, the influence of the annealing on the thermal properties of the PLA films can be summarized as follows:

(1) Annealing was responsible for an increase or decrease of the crystallinity compared to that of raw material. On the other hand, melting temperature and shape of the melting endothermic signal do not seem to be influenced by the annealing.
(2) After the second heating run, a higher tendency to crystallize was recorded for the samples. Similar conclusion was obtained by Carrasco and collaborators [38] on their thermal and mechanical study on the processed poly(lactic acid). In addition, glass transition temperature was approximately the same for raw and annealed materials (T_g_ = 54−58 °C; Δ_cp_ = 0.5−0.6 J/g °C), determined during the second heating scan. The mid-point T_g_ value was not affected by an increase of the crystalline fraction, which act as a physical entanglement, limiting the molecular mobility of amorphous zones. A broadening of the melting transition signal was thus recorded.
(3) A visible cold crystallization exothermic signal was recorded only for annealed samples.
(4) Endothermic shoulder located at about 140 °C was recorded for annealed samples, attributed to a lamellar population generated during heat treatment.
(5) No detectable change in opacity of the films was detected after annealing.

### 2.4. Mechanical Behavior

Food packaging applications put specific demands on the properties of the materials. Besides barrier behavior, the mechanical characteristics such as modulus and elasticity of the material are of decisive importance for the suitability of the materials as packaging. To provide insight into the mechanical properties of the PLA polymer films, tensile measurements and cycling loading tests were performed, to assess the extent of mechanical reinforcement resulting from the surface modification of PLA resin. The results of tensile testing are summarized in Table 7. 

In general, the tensile modulus as a measure of stiffness is improved at the expense of elongation representing the degree of ductility when the reinforcing particles are incorporated into the polymer matrix. In this study, the surface modification of PLA film also caused the decrease in elongation at break of the resultant films, but the extent of reduction was revealed to be slight for PLASiOx film, which may be attributed to easily deformable nature of this molecule in polymer matrix. The extent of reduction was instead remarkable when the PLA film was modified by PVOH and metallization. The tensile properties of elongation at break for the PLASiOx and PLAmet films were measured to very low, demonstrating a low level of ductility. Based on sustainability of elongation property after the surface modification of PLA resins, it is believed that the films analyzed in this study may be utilized for the application of practical packaging film where a required proper level of ductility must be well-defined. All PLA films showed anisotropic behavior, with a lower tensile strength and Young modulus in the *MD* direction than in the *CD* direction.

In particular, although PLA40 is thicker than PLA30, it shows less mechanical strain due to the less crystallinity percentage, with a consequently higher elasticity. For the three other samples, an increase of E modulus, together with σ^y^ and σ^M^ was recorded, showing a strain hardening, induced by an increase of the crystalline portion and by the surface treatment. PLAPVOH and PLA metallized showed the highest tensile strength due to their higher crystallinity. In the same samples, σ^B^ is lower than σ^M^, showing defect on the chemical microstructure [39]. 

**Figure 7:**
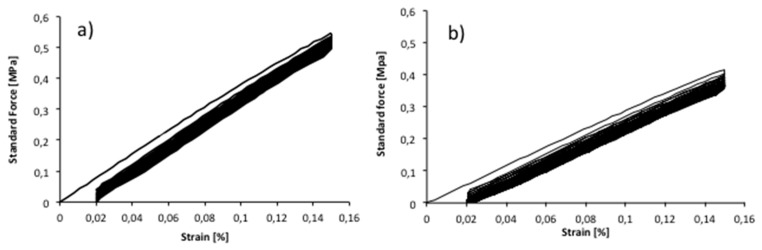
Stress–strain curves of PLAmet sample, upon cycle loading (overlay of all cycles) for: (**a**) CD; and (**b**) MD direction.

The cycle-loading tests demonstrated that the mechanical properties of the copolymers were affected by the repeated loading, especially after the first recorded cycle. In Figure 7, an example of the cycle stress–strain curves of PLA metallized film, in MD and CD direction, strained upon 0.15% of strain are reported. 

This value was chosen in order to perform the test in the elastic region, taking into consideration the ε^y^ recorded value (linear elastic field). The same value was used for all PLA films. The sample was very little elastic, showing ca. 77.5% recovery for MD direction and CD direction, after 25 cycles. A mechanical hysteresis was characteristic of the first cycle resulting in a relatively small initial set. As can be observed in Figure 7, during the cycles, the narrowed hysteresis shape was repeated, producing smaller additional set after each cycle, until the 25 cycles. The stress softening in the ascending curve in the 1st cycle can be caused by a rearrangement in the crystalline micro-phase, formed during the film casting [40]. The hysteresis area decreased starting from the 2nd cycle, showing a less additional set area. The difference between the ascending curves can be attributed to a reorganization of the macromolecules during straining. Other PLA films showed a similar cyclic tensile behavior, with about 80% elastic recovery for both MD and CD direction, after 1 and 25 cycles. It must be underlined that the elasticity is very low for all films and that the cyclic stress–strain measurement was done just to have an idea of the elastic recovery of the polymer matrix after repeated cyclic stress. 

## 3. Materials and Methods 

### 3.1. Materials

Five PLA films were studied without any special preliminary treatments (manipulation or film preparation). All compounds are made of PLA: two were unmodified PLA films of different thickness, while the other three were subject to superficial treatments, in order to change their final properties. The metallization with pure Aluminum was performed under vacuum and under low pressure in order to avoid any external contamination. The low pressure was used to allow the small metallic molecules to reach the film plastic surface without run into air and other gases particles, for a homogeneous and uniform distribution. The SiO_x_ coating was applied by vacuum evaporation. Silicon oxide was firstly sublimated by heating with an electron beam and evaporated as glass vapor. Then, the vapor was condensed on the film plastic surface, forming a very thin glass layer, which was well-distributed by a cooling roll. The thin layer was, in its amorphous state, clear and transparent. PVOH coating was formed by evaporation of a PVOH water solution. PVOH resin was dissolved in hot water (80 °C) and then spread on the PLA film surface. Film sample was dried until the water was evaporated. To remove any residual, the surface was washed with isopropanol. Further details on the specific surface treatments of the PLA film sample are present in literature [12,13].

The films were identified as following:

- PLA30: neat PLA film of 30 μm thickness
- PLA40: neat PLA film of 40 μm thickness
- PLASiO_x_: PLA with SiO_x_ coating
- PLAPVOH: PLA with PVOH coating
- PLAmet: PLA metalized with aluminum coating of less than 1 micron thickness

### 3.2. Permeability Measurement

The permeability determination was performed by a manometric method using a Permeance Testing Device, type GDP-C (Brugger Feinmechanik GmbH, München, Germany), according to ASTM 1434-82 (Standard test Method for Determining Gas Permeability Characteristics of Plastic Film and Sheeting), DIN 53 536 in accordance with ISO 15105-1 and according to Gas Permeability Testing Manual (Registergericht München HRB 77020, Brugger Feinmechanik GmbH). The equipment consists of two chambers between which the film is placed. The amount of gas flowing through the membrane is determined from the pressure variation due to the gas accumulation in the closed receiving chamber. The top chamber is filled with the dry test gases at ambient pressure. The permeation at the bottom chamber of the test specimen is determined by the evaluation of the increase in pressure in the previously evacuated volume. The increase in pressure during the test period is evaluated and displayed by a PC. Fluctuation of the ambient temperature during the test was controlled by special software, with an automatic temperature compensation, which minimizes Gas Transmission Rate (GTR) deviations. The film sample, of approximately 10 × 10 in size, was placed between the top and the bottom of the permeation cell. A film mask was used to cover the rest of the permeation chamber. The GTR value, that is the Rate of the film Gas Transmission, was determined considering the increase in pressure in relation to the time and the volume of the device. The pressure was given by the instrument in (Bar) unit. To obtain the data in kPa, the primary SI units, it is necessary to use the following correction factor: 1 Bar = 100 kPa, according to NIST special publication 811 (NIST 2008) [41]. Time Lag (t_L_), Diffusion coefficient (D) and Solubility (S) of the test gases into the film under study were measured in addition to GTR value. The mathematical relations used are well reported in literature [12,13,14,15,27,42]. The operative conditions were: room condition temperature of 23 °C; gas stream of 100 cm^3^/min; 0% of Gas Relative Humidity (RH); sample area of 78.4 cm^2^. Method A was employed in the analysis, as just reported in the literature [15,27,42,43,44], with evacuation of top/bottom chambers. The following 100% pure food grade gases commonly used for modified atmosphere packaging (MAP technology) were used: O_2_, CO_2_, N_2_, N_2_O, C_2_H_4_, Air (21%O_2_, 79%N_2_), MA (79%N_2_O/21%O_2_). 

For the determination of the activation energies of the permeation process, films were analyzed at a temperature of 5, 10, 20, 23, 30 and 40 °C, using dry gases with 0% RH. 

Chamber and sample temperature were sets by an external thermostat, KAAKE-Circulator DC10-K15 type (Thermoscientific, Selangor Darul Ehsan, Malaysia). 

A gas mixing system Witt-Gasetechnik GmBh & Co KG (Witten, Germany) type Km 100-4 was used to obtain the desired gas flow mixture inside the permeability device.

All experiments were taken in triplicate and a good reproducibility was achieved. The mean value is presented. 

### 3.3. Thickness Determination

The film thickness was determined using the Sample Thickness Tester DM-G, consisting of a digital indicator (Digital Dial Indicator) connected to a PC. The reading was made twice per second (the tool automatically performs at least three readings), measuring a minimum, a maximum and an average value. The thickness value is expressed in μm or inches and the measuring range is from 12.5 μm to 100 μm, with a resolution of 0.001 μm. The reported results represent the mean value thickness of three experimental tests run at 10 different points on the polymer film surface at room temperature.

### 3.4. Differential Scanning Calorimetry (DSC) Measurements

Enthalpies and temperatures of phase changes were determined calorimetrically by using a Perkin-Elmer type Pyris DSC-6 differential scanning calorimeter equipped with a nitrogen liquid sub ambient accessory and calibrated with high purity standards, such as Indium and Tin. Polymer films were cut into small pieces of 2 mm^2^ and placed in a 50 μL sealed aluminum crucibles. Sample mass of 5–10 mg was used. In order to avoid film contamination, special care was taken during handling, working with gloves and tweezers. After isothermally keeping the system for 3 min at a temperature of at −10 °C, weighed samples were first heated, with a heating scanning rate of 10 °C/min, from −10 to 180 °C (first scan) and then, after a further isothermally keeping the system for 3 min at a temperature of 180 °C, were quenched to −10 °C at a cooling rate of 100 °C/min. Finally, after isothermally keeping the system for 5 min, samples were reheated from −10 °C to the same temperature as the first run, at a heating rate of 10 °C/min (second run). 

The same DSC trace experiment was performed after 1 h and 4 h of isothermally keeping the system at 5, 10, 20, 30 and 40 °C (annealing treatment). All experiments were performed under nitrogen flow (20 cm^3^/min). The melting temperature (T_m_) was determined as the peak value of the endothermic phenomena in the DSC curve while the crystallization temperature (T_c_) was determined as the peak value of the isothermal phenomena in the DSC curve. The melting enthalpy (ΔH_m_) of the crystal phase was calculated from the area of the DSC endothermic peak as well as the crystallization enthalpy (ΔH_c_). T_c_ and T_m_ values are well reproducible from the second run, while those relating to the first run are affected by thermal and mechanical history to which the samples were subjected. T_g_ and Δ_cp_ data were obtained from the second run. Calorimetric analysis was performed on film samples in triplicate. 

### 3.5. Tensile Stress–Strain Test and Tensile Cycling Test

Tensile testing of the copolymers was performed using a Zwick Roell Texture machine (Zwick Roell, 2125 Barrett Park Drive, Suite 107, 30144 Kennesaw, GA, USA) mod Z2.5, equipped with a rubber grip and controlled by a computer. A pre-load of 1 MPa was used with a pre-load speed of 5 mm/min. A 500 N load cell was used. Stress–strain and cyclic test measurements were performed on rectangular films of 5 mm wide and 50 mm high, with an initial grip separation of 23 mm. The tensile stress–strain and the cycling loading test measurements were performed with a crosshead speed of 50 mm/min. Twenty-five cycles were recorded at maximum and the film samples were strained up to 0.15%. Five different samples from the same film were tested for each copolymer composition and the results were provided as the average value ± standard deviation. All tests were carried out in accordance with ASTM D638 Standard Test Method for Tensile Properties of Plastics, for film thickness below 100 microns. Films were analyzed in the Cross Direction (CD) and in the Machine Direction (MD), according to literature [13].

## 4. Conclusions

Transition from conventional barrier materials based on petrochemical biostable polymers to natural biodegradable polymer packages as innovative systems requires intensive scientific and industrial explorations. The above research complex can be exploited not only for PLA and its derivatives, but also for the other biodegradable polyesters such as polyhydroxyalkanoates, and copolymers of poly(butylenesuccinate)s and poly(ε-caprolactone). The subsequent advancement in innovative membrane packaging will be devoted to nanobiocomposites with “smart”, stimuli-responsive characteristics and controlled perm-selectivity. 

In the matter of barrier properties, the neat PLA films have shown the GTR values that are lower than the conventional synthetic polymer ones, while the PLA-coated films have a wide variety in gas permeability characterization, changing drastically from a low to a very high barrier material. In the operated range of temperatures, the gas permeability rank mainly corresponds to the following sequence: N_2_O > CO_2_ > MA > O_2_ > N_2_ > C_2_H_4_ ≈ Air.

For the polar gas molecules containing the oxygen group, the GTR values exceed the corresponding values of the nonpolar molecules that can be explained by the predominance of gas solubility contribution into the general process of permeability.

Coating and metallization modify PLA structure especially in the surface layers and hence change such physical chemical characteristics as glass transition temperature, melting temperature, enthalpy relaxation, cold crystallization temperature, etc. An evaluation of its functional properties is essential before its use as alternative material instead of traditional petroleum based packaging materials. This research additionally highlights the importance of gas transmission study in combination with chemical physical polymer behavior to obtain an effective tool for implementation of barrier properties and revealing the packaging critical points. 

## Figures and Tables

**Table 1:** O_2_-GTR data at 23 °C for PLA films sample.

Samples	PLA30	PLA40	PLASiOx	PLAPVOH	PLAmet
GTR	501 ± 0.47	500 ± 0.47	5.47 ± 0.05	23.2 ± 0.05	71.9 ± 0.31
S	5.10 × 10^–2^ ± 3.67 × 10^–3^	7.04 × 10^–2^ ± 1.44 × 10^–3^	-	-	1.42 × 10^–3^ ± 4.11 × 10^–4^
D	3.31 × 10^–8^ ± 2.33 × 10^–9^	3.21 × 10^–8^ ± 6.60 × 10^–10^	-	-	1.20 × 10^–7^ ± 2.96 × 10^–8^
t_L_	42.0 ± 2.94	78.3 ± 1.70	-	-	5.00 ± 1.41
Thickness	29.5 ± 0.29	39.6 ± 0.21	44.9 ± 0.50	28.7 ± 0.50	19.4 ± 0.22

GTR, cm^3^/m^2^ d bar; S, cm^3^/cm^2^ bar; D, cm^2^/s; t_L_, s; thickness, μm.

**Table 2:** Perm-selectivity ratio at 5, 10, 20, 23, 30 and 40 °C.

Sample	CO_2_/O_2_	N_2_/O_2_	N_2_O/O_2_	C_2_H_4_/O_2_	MA/O_2_	Air/O_2_	Air_2_/O_2_
PLA30	1.91/2.04/2.34/	0.14/0.14/0.15	2.63/2.93/3.04	0.04/0.05/0.05	0.97/0.99/1.04	0.14/1.05/1.05	-
	2.37/2.21/2.47	0.12/0.15/0.08	3.12/2.89/3.28	0.06/0.06/0.08	1.18/1.08/2.84	0.17/0.07/0.06	-
PLA40	2.08/2.06/1.93	0.12/0.12/0.13	2.24/2.25/2.46	0.04/0.05/0.04	1.17/0.95/0.95	0.10/0.28/0.52	0.10/0.10/0.94
	2.03/2.15/2.26	0.16/0.16/0.16	2.51/2.67/2.84	0.05/0.05/0.05	0.95/0.94/0.98	0.76/-/-	1.00/0.96/1.10
PLASiO_x_	1.34/1.74/2.10	0.55/0.51/0.40	2.15/2.46/2.83	0.44/0.49/0.37	1.66/1.47/1.36	0.81/0.49/0.37	-
	2.27/2.26/2.41	0.48/0.33/0.34	3.01/2.81/3.39	0.36/0.28/0.25	1.36/1.23/1.02	0.51/0.36/0.41	
PLAPVOH	8.08/6.97/9.97	0.21/0.20/0.18	7.14/14.99/14.35	0.03/0.04/0.04	1.75/1.16/1.19	0.76/0.43/0.33	-
	12.59/10.92/9.66	0.25/0.26/0.23	16.65/12.92/11.67	0.08/0.14/0.14	1.59/1.73/1.87	0.40/0.36/1.50	
PLAmet	7.26/6.22/6.02	7.26/6.22/6.02/	5.54/6.05/6.70	0.14/0.13/0.21	6.27/5.84/5.96/	1.25/1.19/0.24/	-
	5.57/5.44/4.63	5.57/5.44/4.63	5.07/4.99/4.58	0.25/0.26/0.23	5.66/5.95/5.74	0.33/0.43/0.44	

**Table 3:** E_GTR_, H_S_, E_D_ data for pure gases, in the 5 °C to 40 °C temperature range, with the linear regression coefficients *R*^2^ (between brackets).

Samples	E_GTR_ (kJ/mol)	H_S_ (kJ/mol)	E_D_ (kJ/mol)	E_GTR_ (kJ/mol)	H_S_ (kJ/mol)	E_D_ (kJ/mol)
	For O_2_			For CO_2_		
PLA30	21.8 ± 0.26 (0.99)	0.91 ± 0.22 (0.00)	19.1 ± 0.13 (0.57)	26.7 ± 0.31 (1.00)	−9.90 ± 0.32 (0.47)	31.0 ± 0.17 (0.97)
PLA40	20.5 ± 0.12 (1.00)	−11.8 ± 0.10 (0.81)	32.2 ± 0.18 (0.97)	22.2 ± 0.15 (0.98)	0.77 ± 0.05 (0.02)	21.2 ± 0.18 (0.88)
PLASiOx	39.0 ± 0.31 (1.00)	-	-	50.5 ± 0.24 (0.99)	10.2 ± 0.09 (0.05)	39.6 ± 0.21 (0.51)
PLAPVOH	31.9 ± 0.11 (0.95)	-	-	39.2 ± 0.23 (0.96)	–15.8 ± 0.11 (0.45)	55.1 ± 0.18 (0.86)
PLAmet	22.1 ± 0.21 (0.99)	–63.7 ± 0.23 (0.24)	35.6 ± 0.13 (0.42)	13.9 ± 0.12 (1.00)	–10.8 ± 0.07 (0.89)	24.6 ± 0.02 (0.97)
	For N_2_			For N_2_O		
PLA30	12.9 ± 0.13 (0.56)	8.01 ± 0.11 (0.07)	5.14 ± 0.11 (0.03)	25.2 ± 0.21 (1.00)	5.24 ± 0.13 (0.02)	19.9 ± 0.10 (0.25)
PLA40	27.0 ± 0.21 (0.98)	–14.3 ± 0.21 (0.08)	40.4 ± 0.13 (0.79)	25.7 ± 0.22 (1.00)	–24.8 ± 0.21 (0.40)	41.2 ± 0.18 (0.85)
PLASiOx	28.0 ± 0.22 (0.98)	-	-	47.4 ± 0.13 (1.00)	-	-
PLAPVOH	36.2 ± 0.14 (0.97)	22.0 ± 0.01 (1.00)	66.0 ± 0.21 (1.00)	38.0 ± 0.05 (0.76)	–14.5 ± 0.11 (0.49)	82.2 ± 0.12 (0.80)
PLAmet	37.8 ± 0.33 (0.96)	-	-	17.0 ± 0.12 (0.89)	–4.94 ± 0.05 (0.05)	11.0 ± 0.22 (0.06)
	For C_2_H_4_					
PLA30	31.6 ± 0.11 (0.97)	-	-			
PLA40	24.5 ± 0.13 (0.97)	-	-			
PLASiOx	26.7 ± 0.10 (0.98)	-	-			
PLAPVOH	66.7 ± 0.11 (0.97)	-	-			
PLAmet	36.6 ± 0.14 (0.95)	-	-			

**Table 4:** E_GTR_, H_S_, E_D_ data for Air and MA, in the 5 °C to 40 °C temperature range, with the linear regression coefficients (*R*^2^).

Samples	E_GTR_ (kJ/mol)	H_S_ (kJ/mol)	E_D_ (kJ/mol)	E_GTR_ (kJ/mol)	H_S_ (kJ/mol)	E_D_ (kJ/mol)
	For Air			For MA		
PLA30	–18.0 ± 0.01 (0.10)	34.6 ± 0.15 (0.23)	15.2 ± 0.13 (0.10)	39.8 ± 0.11 (0.89)	45.8 ± 0.04 (0.87)	16.5 ± 0.31 (0.97)
PLA40	89.7 ± 0.15 (0.97)	131 ± 0.05 (0.75)	28.9 ± 0.09 (0.25)	17.8 ± 0.09 (0.95)	9.73 ± 0.03 (0.15)	–6.40 ± 0.44 (0.01)
	78.2 ± 0.21 (0.86)	77.3 ± 0.08 (1.00)	-			
PLASiOx	26.4 ± 0.16 (0.84)	-	-	30.1 ± 0.21 (0.99)	-	-
PLAPVOH	41.9 ± 0.22 (0.65)	-	-	37.1 ± 0.13 (0.98)	–176 ± 0.44 (1.00)	145 ± 0.19 (1.00)
PLAmet	–3.62 ± 0.21 (0.01)	44.8 ± 0.21 (0.67)	–28.4 ± 0.21 (0.38)	20.8 ± 0.21 (1.00)	22.3 ± 0.13 (0.41)	–8.86 ± 0.09 (0.10)

**Table 5:** DSC data for the thermal transitions observed on PLA film samples.

Sample	1st Scan			2nd Scan								
	T_monset_ (°C)	T_m_ (°C)	ΔH_m_ (J/g)	T_gonset_ (°C)	T_g_ (°C)	Δcp (J/°C g)	T_conset_ (°C)	T_c_ (°C)	ΔH_c_ (J/g)	T_monset_ (°C)	T_m_ (°C)	ΔH_m_ (J/g)
PLA30	134 ± 0.3	147 ± 0.1	19 ± 0.2	54 ± 0.2	54 ± 0.1	0.5 ± 0.1	115 ± 0.3	128 ± 0.1	1.4±	146 ± 0.3	149 ± 0.2	1.5 ± 0.0
PLA40	139 ± 0.2	146 ± 0.1	16 ± 0.1	54 ± 0.2	55 ± 0.1	0.5 ± 0.3	-	-	-	145 ± 0.2	149 ± 0.2	0.7 ± 0.2
PLASiO_x_	136 ± 0.3	146 ± 0.2	18 ± 0.1	54 ± 0.3	55 ± 0.1	0.5 ± 0.1	-	-	-	145 ± 0.2	148 ± 0.1	0.4 ± 0.0
PLAPVOH	139 ± 0.2	147 ± 0.1	18 ± 0.1	54 ± 0.1	55 ± 0.1	0.6 ± 0.1	124 ± 0.2	129 ± 0.1	0.5±	146 ± 0.2	149 ± 0.2	1.2 ± 0.1
PLAmet	143 ± 0.1	150 ± 0.1	24 ± 0.3	57 ± 0.1	58 ± 0.2	0.5 ± 0.2	116 ± 0.1	129 ± 0.1	4.5±	147 ± 0.1	152 ± 0.1	3.6 ± 0.1

**Table 6:** Crystallinity percentage calculated on standard sample and after 1 h/4 h of isothermally keeping the samples at different temperatures at different temperature.

Sample	Xc (%) Standard	Xc (%) iso at 5 °C	Xc (%) iso at 10 °C	Xc (%) iso at 20 °C	Xc (%) iso at 30 °C	Xc (%) iso at 40 °C
1^st^ scan						
PLA30	20 ± 0.1	15 ± 0.1/19 ± 0.1	20 ± 0.1/22 ± 0.3	20 ± 0.0/18 ± 0.2	21 ± 0.1/21 ± 0.3	20 ± 0.3/15 ± 0.0
PLA40	17 ± 0.1	19 ± 0.2/24 ± 0.1	19 ± 0.1/17 ± 0.1	22 ± 0.0/27 ± 0.2	25 ± 0.3/23 ± 0.2	22 ± 0.2/26 ± 0.0
PLASiO_x_	19 ± 0.0	18 ± 0.1/17 ± 0.2	19 ± 0.0/19 ± 0.0	19 ± 0.0/20 ± 0.0	19 ± 0.3/20 ± 0.4	18 ± 0.1/18 ± 0.1
PLAPVOH	20 ± 0.0	20 ± 0.1/20 ± 0.0	18 ± 0.0/20 ± 0.2	22 ± 0.1/21 ± 0.2	20 ± 0.0/22 ± 0.0	19 ± 0.2/20 ± 0.2
PLAmet	26 ± 0.2	23 ± 0.2/22 ± 0.3	27 ± 0.2/24 ± 0.0	26 ± 0.1/22 ± 0.1	25 ± 0.3/16 ± 0.4	23 ± 0.2/24 ± 0.2
2^nd^ scan						
PLA30	0.1 ± 0.1	1.0 ± 0.2/1.3 ± 0.1	3.0 ± 0.0/2.3 ± 0.0	2.8 ± 0.1/1.3 ± 0.0	1.6 ± 0.1/0.9 ± 0.0	-/1.5 ± 0.0
PLA40	0.8 ± 0.1	0.8 ± 0.1/1.0 ± 0.1	0.5 ± 0.0/0.8 ± 0.1	-/1.1 ± 0.0	5.3 ± 0.1/0.8 ± 0.1	0.5 ± 0.1/4.6 ± 0.2
PLASiO_x_	0.4 ± 0.0	0.3 ± 0.1/0.4 ± 0.1	0.5 ± 0.1/0.6 ± 0.2	0.8 ± 0.1/0.2 ± 0.2	-/0.5 ± 0.0	0.3 ± 0.0/0.4 ± 0.0
PLAPVOH	0.8 ± 0.0	0.8 ± 0.1/0.9 ± 0.0	0.2 ± 0.1/0.3 ± 0.0	0.5 ± /0.13.8 ± 0.2	0.9 ± 0.0/1.9 ± 0.1	0.8 ± 0.0/0.6 ± 0.1
PLAmet	-	-/1.5 ± 0.0	2.8 ± 0.1/1.6 ± 0.0	2.3 ± 0.0/2.4 ± 0.2	2.8 ± 0.2/2.8 ±0.1	-/2.3 ± 0.1

**Table 7:** Tensile properties of PLA films.

Polymer	Young’s Modulus (E_t_) (GPa)	Yield Strength (σ^y^) (MPa)	Yield Strain (ε^y^) (%)	Tensile Strength (σ^M^) (MPa)	Strain at Tensile Strength (ε^M^) (%)	Stress at Break (σ^B^) (MPa)	Strain at Break (ε^B^) (%)	Work up to Break (W_B_) (Nmm)	Energy Density up to Break (W_B_/V) (MJ/m^2^)
PLA30micron	3.0 ± 0.3/	79.5 ± 11.9/	0.41 ± 0.01/	79.5 ± 11.9/	0.41 ± 0.01/	67.0 ± 11.2/	17.2 ± 3.5/	38.2 ± 11.2/	114 ± 33.5/
MD/CD	3.3 ± 0.4	80.2 ± 14.2	0.3 ± 0.01	80.2 ± 14.2	0.32 ± 0.02	80.2 ± 14.2	0.32 ± 0.02	4.6 ± 0.9	1.7 ± 0.4
PLA40micron	2.0 ± 0.1/	66.7 ± 5.0/	0.44 ± 0.04/	71.8 ± 3.5/	16.7 ± 0.16/	71.8 ± 3.5/	16.7 ± 0.16/	41.9 ± 1.6/	93.1 ± 3.6/
MD/CD	3.0 ± 0.1	75.9 ± 3.6	0.37 ± 0.02	95.8 ± 15.8	5.8 ± 1.3	95.8 ± 15.8	5.8 ± 1.3	18.9 ± 6.1	42.1 ± 1.5
PLASiOx	2.5 ± 0.2/	80.3 ± 4.8/	0.50 ± 0.00/	80.3 ± 4.8/	0.5 ± 0.00/	36.9 ± 2.2/	13.0 ± 0.4/	27.7 ± 4.5/	53.3 ± 8.6/
MD/CD	3.5 ± 0.2	95.9 ± 4.9	0.34 ± 0.02	117.1 ± 12.9	5.2 ± 0.6	100.2 ± 32.1	5.8 ± 0.3	26.0 ± 2.4	50.2 ± 4.5
PLAPVOH	2.9 ± 0.1/	90.3 ± 3.9/	0.46 ± 0.02/	90.3 ± 3.9/	0.46 ± 0.02/	71.6 ± 3.7/	4.1 ± 1.9/	9.2 ± 4.8/	29.4 ± 14.4/
MD/CD	3.7 ± 0.3	108.1 ± 10.1	0.46 ± 0.11	108.1 ± 10.1	0.46 ± 0.11	89.0 ± 9.5	2.1 ± 0.8	6.0 ± 2.3	17.9 ± 6.9
PLAmetallised	3.0 ± 0.1/	85.4 ± 5.3/	0.34 ± 0.03/	85.4 ± 5.3/	0.36 ± 0.03/	82.3 ± 3.5/	0.39 ± 0.07/	0.4 ± 0.1/	2.0 ± 0.5/
MD/CD	4.0 ± 0.1	105.8 ± 3.1	0.34 ± 0.01	141.2 ± 4.7	6.6 ± 0.4	141.2 ± 4.7	6.6 ± 0.4	14.9 ± 1.0	68.0 ± 4.3
